# Case Report: Application of Mixed Reality Combined With A Surgical Template for Precise Periapical Surgery

**DOI:** 10.3389/fsurg.2022.923299

**Published:** 2022-06-10

**Authors:** Tingting Jia, Bo Qiao, Yipeng Ren, Lejun Xing, Baichen Ding, Fang Yuan, Qiang Luo, Hongbo Li

**Affiliations:** ^1^ Department of Stomatology, The First Medical Centre, Chinese PLA General Hospital, Beijing, China; ^2^ Department of Oncology, The Fifth Medical Centre, Chinese PLA General Hospital, Beijing, China

**Keywords:** MR, 3D printed templates, apical microsurge, chronic apical periodontitis, prognosis, diagnose

## Abstract

**Objective:**

The etiology of apical diseases is diverse, and most are due to incomplete root canal therapy. The common clinical manifestations include gingival abscess, fistula and bone destruction. The currently existing limitation of procedures is that surgeons cannot visually evaluate the surgical areas. We sought to combine mixed reality (MR) technology with a 3-dimensional (3D) printed surgical template to achieve visualization in apical surgery. Notably, no reports have described this application.

**Methods:**

We created visual 3D (V3D) files and transferred them into the HoloLens system. We explained the surgical therapy plan to the patient using a mixed reality head-mounted display (MR-HMD). Then, the 3D information was preliminarily matched with the operative area, and the optimal surgical approach was determined by combining this information with 3D surgical guide plate technology.

**Results:**

We successfully developed a suitable surgical workflow and confirmed the optimal surgical approach from the buccal side. We completely exposed the apical lesion and removed the inflammatory granulation tissue.

**Conclusion:**

We are the first group to use the MR technique in apical surgery. We integrated the MR technique with a 3D surgical template to successfully accomplish the surgery. Desirable outcomes using minimally invasive therapy could be achieved with the MR technique.

## Introduction

The synchronization of a patient’s body with surgical equipment and images is important for minimally invasive surgery. Surgeons need to be able to better associated 2-dimensional (2D) images with 3-dimensional (3D) space. This approach will allow more precise and minimally invasive surgical procedures. At present, there are many methods to combine images with objects, which can enhance the representativeness of medical images of patients (1). However, despite the advances in medical imaging technology, medical images are still unable to be seamlessly integrated with surgical procedures (2). Stereo vision technology has been widely used to solve this problem, and some progress has been made, such as wearable computer technology (3).

Traditional stereo vision technologies include augmented reality (AR) and virtual reality (VR). Since the early 1990s, VR and AR technologies have been dedicated to meeting the needs of 3D visualization in medical diagnostics (4). VR technology has been widely used in medical education, surgical planning, and extensive treatment intervention (4). In this context, AR not only provides real environmental information but also provides virtual information, which improves the perception of reality and makes the technology more widely applicable (5). Currently, due to the intricate anatomy of the maxillofacial region, the application of AR technology in oral surgery has received increasing attention (6, 7). Additionally, traditional diagnostic imaging techniques, such as X-ray, computed tomography (CT), magnetic resonance imaging and angiography, can also be a good source of AR information (8).

Although AR and VR devices have shown broad prospects, certain significant limitations exist. First, AR technology cannot interact with 3D packets; in addition, VR technology will eliminate real-world environments. Many researchers have strived to find ways to integrate VR and AR, and thus mixed reality (MR) technology was developed, which combines the advantages of VR and AR to provide practical help to surgeons (3). MR technology simulates 3D images, reducing the deviation between operating space and visualization (1). Currently, MR technology is being applied to visceral surgery (1), oral implant surgery (3), and congenital heart disease (9) diagnosis and has achieved success.

Periradicular curettage is a part of the treatment procedure of periradicular surgery. Its main purpose is to remove pathological periradicular tissues to increase visibility and accessibility and facilitate the treatment of the apical root canal system or, in some cases, for the removal of harmful foreign materials present in the periradicular area (10). For the teeth with intact cortical bone on the lip and buccal side, the positioning of the apical and lesion areas and accurate deboning have always been difficult points in periradicular surgery (11). The traditional surgical operation involves determining the surgical approach based on experience. This method is more difficult for beginners and can easily increase the damage to the bone tissue, miss the infected root canal, and prolong the healing time of the wound. At present, the use of cone beam computed tomography (CBCT) imaging to create a 3D surgical template to guide periapical surgery can greatly improve the intraoperative accuracy (12). However, the surgical template can only identify the position of the approach point before the operation and cannot reveal important anatomical structures around the operation area. Therefore, to achieve minimally invasive and precise treatment and to reduce the technical sensitivity, this study used an MR assistance combined with a 3D-printed surgical template to perform apical surgery and explored its clinical feasibility, range of application and clinical efficacy.

## Methods

### Construction of the Surgical Template

A gypsum model of the mandibular dentition was optically scanned and output in stereo-lithography (STL) format. Additionally, CBCT data were output in DICOM format. We imported the above two sets of data into the 3Shape software program to ensure that they overlapped and matched. After the template design was completed, the 3D resin printer created a surgical template.

### Patient General Information

We defined the tooth number according to the guidelines of the FDI World Dental Federation.

**Figure 1:**
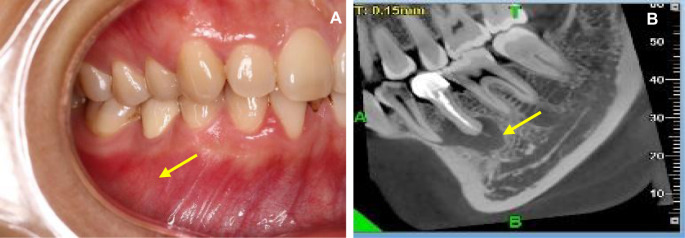
Buccal image and CBCT (NewTom 5G Version FP) examination. (**A**) Buccal image of the affected tooth. (**B**) CBCT image of the lesion area.

Case: patient, female, 27 years old. Chief complaint: gingival swelling in the mandibular posterior teeth for 1 year. Clinical examination: tooth 45: porcelain crown restoration, edge tightness, percussion pain (++), looseness, no swelling or peripyema on the gingival margin, and no fistula on the buccal gum. CBCT (NewTom 5G Version FP) showed 3–4 mm of gutta-percha beyond the apical foramen. The apical area had a wide range of low-density shadows, and the boundary was clear. The buccal and lingual cortices were continuous and intact, and the mental foramen was located below the tip of tooth 45, which was close to the periapical lesion (Figures 1A,B).

### Development of the Surgical Plan Using MR

**Figure 2:**
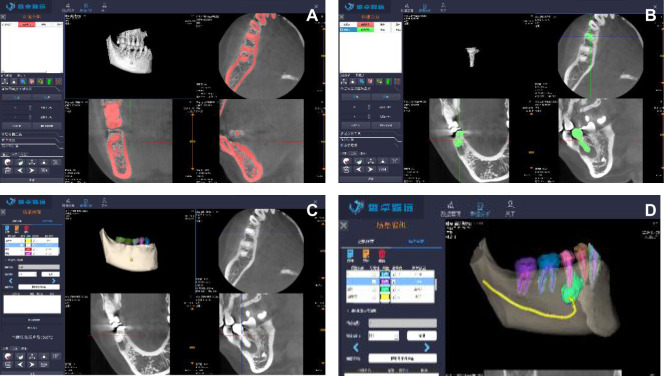
The 3D model of the patient. (**A**) Rending the target area (**B**) Obtaining the mandible and the affected tooth (**C**) Obtaining the lesion area (**D**) Defining the surface color of the V3D model.

To convert the 2D images into 3D format for visualization via the mixed reality head-mounted display (MR-HMD) and to optimize this 3D model for intraoperative application, many relevant computer software programs were needed to process information during the work flow. We used self-developed work station software and the MR system to construct the 3D model of the patient. We first exported a DICOM file containing the original CT data of the patient. Using the software, we changed the gray value to segment the mask of the targeted root region of the right mandibular jaw lesion in the 2D image (Figure 2A). The masks of the teeth and jaws were obtained by the threshold segmentation method, and then the STL model of the bone and teeth was obtained using a Marching Cube algorithm for the mask data (Figures 2B,C). Then, we use our self-developed work station software to import these STL models and specified their color and transparency, and then the files were exported to a holographic case scene in visual 3D (V3D) file format. Finally, we determined the relationships between the lesion area and the adjacent root and inferior alveolar nerve. Different anatomical structures were color-coded on the V3D model; the lower alveolar nerve was marked yellow, and the lesion area was marked green (Figure 2D). In the traditional CBCT image, the lesion area was very close (approximately 2 mm) to the inferior alveolar nerve, but the relationship between the mental foramen and the lesion area was not clear. In the MR image, the relationship between the mental nerve and the green lesion area could be clearly seen.

### Application of the MR-HMD for Surgery

Next, we imported the V3D files created for the operation into the client operating software (Visual 3D, StarNav V1 .0 Beta, Beijing, China). Then, we used the StarNav operating software to upload the V3D files and transfer them to the server. Then, the app in the HoloLens was started, and the holographic case scenario was shown.

**Figure 3:**
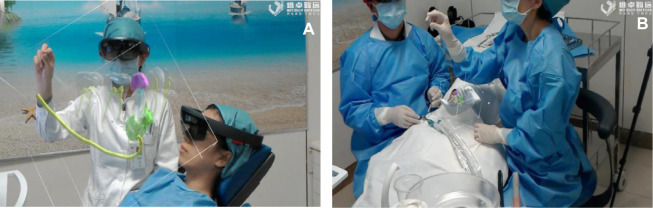
Both the surgeon and the patient can see the surgical field. (**A**) Preoperative communication (**B**) Preoperative matching.

Before the surgery, the assistant surgeon placed the magnetic field emitter within 1 0 centimeters of the patient’s head. The V3D model of the surgical area was wirelessly transmitted into the HoloLens worn by the surgeon and patient. This system enables 3D objects to be steadily positioned relative to operating room surfaces, and it allows objects to be moved, resized and rotated based on the horizontal axis. The maximum texture resolution is 1,268 × 720 pixels, and the system can handle up to 300,000 vertices. The surgeon can adjust the wheel in the headband for their comfort. Both the surgeon and patient can clearly see the surgical region. Most notably, the surgeon can adjust the angle of surgical region to communicate detailed information to the patient (Figure 3A). The V3D data were customized to the patient’s mandible to guarantee accuracy of the surgery (Figure 3B).

### MR Combined With a Surgical Template for Apical Surgery

**Figure 4:**
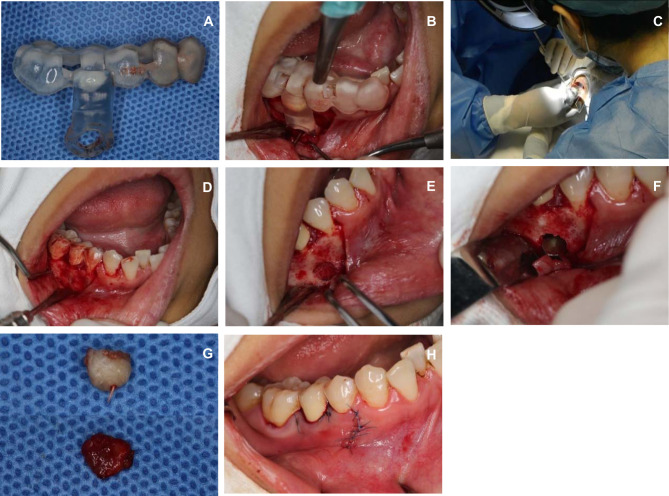
The surgical template and the MR holographic image were combined to position the mandibular opening window. (**A**) Intraoperative surgical template application (**B**) Retaining surgery template (**C**) Projecting MR holographic image into patients (**D**) Precise positioning of the window midpoint (**E**) Opening the window on the bone surface (**F**) Cutting off apical lesions (**G**) Apical lesions were successfully removed (**H**) Tightly sutured mucoperiosteal flap.

A one-time root canal therapy for tooth 44 was completed one week prior to the surgery. The surgical template was routinely sterilized for intraoperative use (Figure 4A). The area was routinely disinfected and covered with towels, and compound antica anesthesia was administered for local infiltration. A vertical incision was made in the middle of tooth 44. An incision was made in the sulcus from tooth 44 to tooth 46. The flap was inverted, and the buccal bone plate was exposed. No obvious bone opening window was observed. The surgical template was placed in a predetermined position to help determine the center point of the window opening. At the same time, the doctor was wearing HoloLens Glasses, which projected the MR hologram onto the patient’s lesion. In this manner, the surgical template and the MR holographic image were combined to position the mandibular opening window, which ensured the accuracy of the window opening position (Figures 4B–D). Because the image could not realize real-time positioning, the HoloLens MR-HMD was not worn during the operation. Surgical tools were used to open a 4 mm*4 mm window at the location of the bone surface (Figure 4E), the apical lesion area was exposed, and 3 mm of the root tip was cut to form a complete plane. After the root tip was removed, it could be seen that the root canal was over filled, and the inflammatory granulation tissue was large (Figures 4F,G). The granulation tissue of the apical area was completely removed. Toluidine blue staining of the root tip was not observed under a microscope, and no obvious crack was found. An ultrasonic tip (KiS Tips, Obtura Spartan, USA) was used to prepare a canal 3 mm deep along the long axis of the root. Then, 0.9% sodium chloride was injected, the root canal was wiped dry with a paper tip, and a mineral agglomerate was inserted (ProRootMTA, Dentsply, United States). The root tip was filled. After trimming the surface of the filling area and checking the bone cavity for free foreign matter, 0.9% sodium chloride was used to rinse the apex and bone cavity. The mucoperiosteal flap was repositioned and attached using a 6-0 silk thread (Figure 4H). After the operation, a cotton ball was applied for 30 min to stop bleeding, and the patient was directed to use chlorhexidine mouthwash for 7 days.

**Figure 5:**
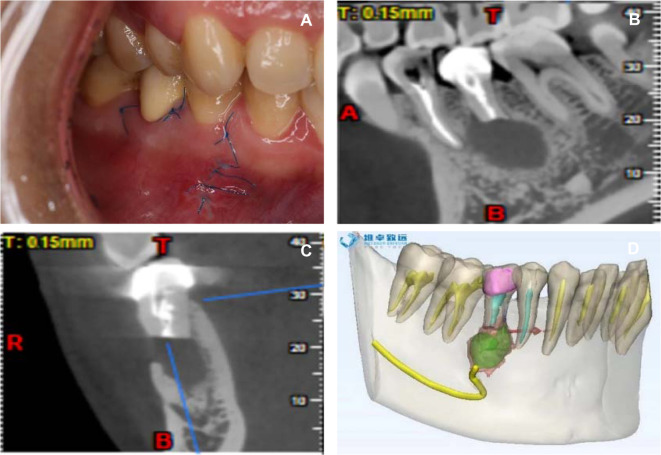
The CBCT images were reviewed. (**A**) One week postoperative wound condition (**B**) Complete resection of apical lesions (**C**) Distance from the inferior alveolar nerve canal in the lesion area after surgical resection (**D**) Resection range covers the extent of the lesion.

After one week, the gums had healed well, the line was removed, and the CBCT images were reviewed. The patient had no numbness in the lower lip and oral mucosa (Figure 5A). According to an image of the apical area reconstructed by postoperative CBCT, the lesion area had been entirely resected (Figure 5B), but a gap between the area and the inferior alveolar neural tube was present (Figure 5C), which was why the patient did not have numbness after surgery. The preoperative and postoperative MR holographic images were superimposed. In the superimposed image, the preoperative lesion area is displayed in green, and the postoperative are is displayed is red; the green area is completely covered by the red area (Figure 5D), indicating that the inflammatory tissue in the operative area was completely removed.

## Discussion

Apical surgery is a common operation in oral clinics, but the surgery success depends mainly on working experience (13). This procedure poses a challenge for young doctors. In the case of a tooth with a relatively small apical lesion accompanied by an intact lip/buccal cortical bone, accurately locating the apical and lesion is extremely important (14). Moreover, clinicians should focus on the location, shape, size, direction and depth of bone removal. In the conventional process of deboning and window opening, the position of the apex is generally determined according to the anatomical shape of the root and the preoperative X-ray (15). Generally, excess apical region bone would be removed based on the evidence mentioned above. Such an operation will also increase the damage to the bone tissue, prolong the healing time of the wound, and easily produce intraoperative and postoperative complications.

The development and application of new equipment and materials have completely changed the mode of periapical surgery, making periapical surgery more minimally invasive and accurate and improving the outcomes (11). To accurately locate the approach for periapical surgery, it is common to combine CBCT images before surgery to create a surgical template. However, the current surgical templates still have the following limitations. First, the template can only identify the location of the surgical approach and cannot reveal the 3D relationship between the complete field and important anatomical structures. In addition, since the template needs to occupy the surgical space, the template can only be used for preoperative point positioning. When the positioning point is determined, the surgical template needs to be removed, and the operation is performed under the normal surgical field of view. Real-time spatial positioning and manipulation of the operation area cannot be realized. Therefore, we introduced MR technology to solve these problems and optimize periapical surgery.

In this case, the patient had good integrity of the buccal bone plate and no clear apical fistula. Before surgery, we used a combination of MR and a surgical template to determine the surgical approach. Since the holographic 3D image generated by MR can be combined with the actual situation in the patient’s mouth, the surgical guide is positioned more accurately. In addition, we invited patients to wear HoloLens Glasses in parallel with the doctor before surgery and explained to the patient the cause, harm and complications of apical lesions and the specific process of the operation. The patient expressed full understanding of the information based on the holographic 3D images. After such effective communication, the patient expressed a great relief of nervousness and was confident in the success of the operation. The patient cooperated well during the entire process of the operation, experienced no discomfort during the postoperative recovery period, and actively cooperated with the doctor to obtain image data. After the operation, we performed repeat CBCT and generated MR images. Comparison of MR images before and after surgery showed that the lesion area (green part) was completely surrounded by the postoperative cavity area (red part, Figure 5D).

Although MR technology was successfully applied in this case of apical surgery, it still has limitations. The current MR method still lacks real-time imaging capability (16), and it is impossible to achieve real-time image matching during surgery (17). When the patient’s body position moves, the image will deviate from the actual situation. Therefore, in this case, after positioning point marking was completed, the HoloLens Glasses worn by the surgeon and the surgical template were removed, and conventional surgery was performed (18). A key direction of future research will be how to accurately integrate the holographic 3D image with the actual anatomy of the human body in real time (16, 19). Additionally, we also look forward to combining MR technology with navigation technology to achieve true visualization, digitization and precision. Furthermore, this technology can be extended to surgery with high-precision visual navigation requirements such as oral implant surgery and maxillofacial reconstruction surgery (20). We firmly believe that this new method may bring changes to the field of oral surgery.

## Data Availability

The original contributions presented in the study are included in the article/Supplementary Material, further inquiries can be directed to the corresponding author/s.
